# Warming the postpartum body as a form of postnatal care: An ethnographic study of medical injections and traditional health practices in Cambodia

**DOI:** 10.1371/journal.pone.0228529

**Published:** 2020-02-06

**Authors:** Alessandra N. Bazzano, Jeni A. Stolow, Ryan Duggal, Richard A. Oberhelman, Chivorn Var

**Affiliations:** 1 Department of Global Community Health and Behavioral Sciences, Tulane School Public Health and Tropical Medicine, New Orleans, LA, United States of America; 2 Tulane University School of Medicine, New Orleans, LA, United States of America; 3 Reproductive Health Association of Cambodia, Phnom Penh, Cambodia; 4 National Institute of Public Health, Phnom Penh, Cambodia; Georgia Southern University, UNITED STATES

## Abstract

Postpartum care is a critical element for ensuring survival and health of mothers and newborns but is often inadequate in low- and middle-income countries due to barriers to access and resource constraints. Newly delivered mothers and their families often rely on traditional forms of postnatal care rooted in social and cultural customs or may blend modern and traditional forms of care. This ethnographic study sought to explore use of biomedical and traditional forms of postnatal care. Data were collected through unstructured observation and in-depth interviews with 15 mothers. Participants reported embracing traditional understandings of health and illness in the post-partum period centered on heating the body through diet, steaming, and other applications of heat, yet also seeking injections from private health care providers. Thematic analysis explored concepts related to transitioning forms of postnatal care, valuing of care through different lenses, and diverse sources of advice on postnatal care. Mothers also described concurrent use of both traditional medicine and biomedical postnatal care, and the importance of adhering to cultural traditions of postnatal care for future health. Maternal and newborn health are closely associated with postnatal care, so ensuring culturally appropriate and high-quality care must be an important priority for stakeholders including understand health practices that are evolving to include injections.

## Background

Postnatal care is recognized as an important aspect of improving neonatal and maternal survival [1, 2], but is often overlooked in provision of health services [3]. The period after birth is the most vulnerable one for both mothers and their newborns. Gaps in postnatal care have been identified as an important barrier to improving the continuum of care for improving maternal and newborn health [4]. In Cambodia, maternal mortality has decreased markedly [5], but gaps in equity persist and quality of care remains an important priority [4, 6, 7].

In Cambodia, approximately 75% of women attend maternity care services provided by a skilled attendant [8]. Most seek care through public health facilities, which is provided by primary or secondary midwives during the antenatal period, delivery, and postnatally, requiring modest user fees set by the local operational health district, or provided free for those deemed unable to pay [5, 8, 9]. A robust culture of traditional medicine (only available through the private sector) also greatly influences care during the perinatal period. As is the case in many countries, supplies and medicines needed for perinatal care from public health facilities must be paid out of pocket, along with transportation, food, and accommodation [10]. During antenatal visits, women have blood pressure measured, are counseled on various health topics including general advice on a healthy diet, breastfeeding, and signs of complications requiring care seeking [11].

Given the importance of local understandings of health and illness in the perinatal period, several qualitative studies have focused on Cambodian mothers’ care behaviors in relation to traditional medicine before and after childbirth [12–16]. Traditional medicine is considered an important component of health care for Cambodian consumers, with estimates from the Ministry of Health (MOH) of 40–50% of the population reportedly using traditional medicine on a regular basis [17]. Recently the use of traditional medicine in Cambodia has come under scrutiny for its impact on out of pocket expenditures, which can lead to impoverishment among lower income populations where the practice of using traditional medicine is very common [18].

Traditional maternal medicine in Cambodia is influenced by Khmer etiology and by the relationship of hot and cold states to health. A woman is considered to be in a hot state during pregnancy, loses heat through the birthing process, and is considered in a dangerously cold state afterwards. The latter condition is associated with vulnerability throughout the postpartum period referred to locally as *sor sai kchey* which conveys the meaning that “the body is weak”. The individual words *sor sai* can be translated to “vessels” or “small tubes that allow flow” and the word *kchey* may been translated as “weak” or “green/not yet mature”. If heat is not returned during the post-partum period, illnesses or more severe symptoms may extend from this state of bodily weakness into future illness over the lifetime, referred to as *toa (*sometimes spelled *tos)*, which directly influence post-partum care practices and behaviors [4–5]. Traditional postpartum care is thus focused on restoring “heat” through various means including foods considered to generate heat, tonic beverages containing herbs and/or alcohol, placing warm stones on the abdomen, and other physical means of heating of the body [13, 19]. Heating of a postpartum mother in previous times typically consisted of *ang pleung* or “roasting”. This entailed a newly delivered woman resting on a raised wooden bed over charcoal embers where heating would take place for three to seven days, and the mother would not leave the heated area during the process. Heating the body is thought to help *chha-an sor sai* which may be translated as to “mature/harden” or “cook” the “vessels”. This is commonly understood as the means to prevent post-birth illness, help women recover from childbirth, and restore internal hot/cold balance [12, 13, 20, 21].

A modern, biomedical approach that has become common in the last decade for heat balance is the use of medical injections in the postnatal period to warm the body and promote recovery. However, a gap exists in the literature on these as used for post-partum care, and health consequences as a result of their use. Injections for varied health ailments are usually delivered privately, paid for out of pocket by families, and typically include vitamins, antibiotics, and/or pain medication given in a patient’s home [22]. These injections appear to be typically provided privately by midwives in the homes of women during the post-partum period. Due to the physical sensations associated with post-natal injections, these have been called “heat injections” [13], and have been reported in popular media since as early as 2004 as a replacement for *ang pleung* [23, 24]. However, medical injections generally are often unnecessary, expensive, and raise the risk of blood-borne infections resulting from administration in unregulated settings with potentially unsterile equipment or improper injection practices. Cambodia has one of the highest rates of overall medical injection usage worldwide, where more than one in three individuals reported receiving a medical injection in the previous 12 months, with 65% of those medical injections taking place in the home [25]. However, the study did not identify the specific uses of those medical injections, so the proportion of those which were post-natal is unclear.

There is a paucity of biomedical literature on the use of medical injections for post-partum care, an apparently widespread health practice in Cambodia and for the diaspora [26], thus the current study sought to explore this topic through ethnographic research to understand mothers’ experiences and provide information that may be used to continue improving care that addresses maternal morbidity and mortality. Safety of injection practices has been noted as an important issue for preventing blood-borne illnesses, particulary in lower income countries [27]. As Cambodia continues to experiences rapid economic and social development, this data may inform approaches to improving quality of care, and allow for understanding of the integration of locally acceptable health interventions with biomedical practice.

## Methods

An ethnographic approach was utilized to document and describe routine postnatal care behaviors in the natural setting of communities and homes where families go about day to day activities. Qualitative data collection took place in Takeo, Cambodia from June—August 2016 and included information drawn from 15 in-depth interviews conducted with female participants who had a child under 6 months old and unstructured, opportunistic observation in and around homes as allowed by participants. Maximum variation in participant characteristics guided the purposive sampling approach [28] to include participants in more and less rural areas, and with varying socioeconomic status and educational attainment, and the number of interviews was determined by availability and comfort of interview participants in discussing post-natal care [29]. All interviews were conducted by two of the study authors (AB an experienced qualitative research, RD a junior researcher), alongside Cambodian research colleagues (CV and DK in acknowledgements section) who provided input and translation. All researchers participated in preparation and training for the data collection process, and supervisory critical input was provided by the senior author (CV), a Cambodian physician specialized in reproductive health. Interviews were conducted in homes of participants in Khmer language with translation to English. Interviewees had previously participated in a newborn health intervention [30] with infants up to one week old, which did not specifically address postnatal care of the mother, and those with an infant less than 6 months of age were invited separately to participate in the study using study-specific informed consent procedures and with detailed information provided on the qualitative study aims and procedures. After agreeing and providing written informed consent, the participants engaged in interviews lasting no more than 45 minutes. Potential participants were introduced to the researchers through local community-based staff including village health workers and study coordinators. Discussion guides used for data collection were developed and pre-tested in the study area and translated to and from English for proofing. Interviews were audio recorded at time of interview and field notes were written during review of audio recordings and after interview. All transcripts and notes from ethnographic observation were uploaded to NVivo software in English for the analysis process. Approximately 40 hours of unstructured observation also contributed to field notes and memos included in the analysis. The COREQ guidelines served as a reference for conducting and reporting this research [31].

Written informed consent was provided by all participants, who were provided with information on the study purpose and how data would be used. Precautions were taken to ensure privacy so that interviews took place in a private location, and pseudonyms were used for all participants. The study protocol was approved by the National Ethics Committee Health Research of the Cambodia Ministry of Health and by the Institutional Review Board of Tulane University. The larger trial in relation to which this research was conducted is registered with ClinicalTrials.gov, number NCT02271737. All study data was kept under secured and password protected conditions and only the study authors had access to the data, which is maintained by the National Institute of Public Health in Cambodia.

Qualitative data was synthesized via thematic content analysis [32]. This method utilized content analysis to understand participant’s perspectives. Data analysis steps consisted of: (1) familiarization with transcripts and fieldnotes; (2) iterative development of a coding framework; (3) coding all transcripts; (4) discussion of preliminary themes; and (5) synthesis and interpretation of findings. NVivo software was utilized to facilitate systematic and rigorous exploration of the data. Ethnographic observations around maternal care practices in the home, where interviews also took place, provided contextual information in which to ground the interview data. Photographs were taken during observation of objects and materials used for postpartum care (e.g. herbs, stones, food considered to bring heat to the body), which provided reference material for analysis. These photos were also entered to NVivo and coded to appropriate nodes. Once the preliminary analysis was complete, the research team reflected on how implementation and outcomes reflected textual data collected by the research team, and member checking through one author’s network (CV) in Cambodia facilitated trustworthiness. Major and minor themes related to postnatal care practices were explored to understand behaviors and beliefs.

## Results

Participant characteristics are summarized below in Table 1. Of the fifteen women interviewed, most were between the ages of 25 to 30. Most women had 5–10 years of education. Twelve women interviewed reported at least one type of occupation. All women reported being Buddhist and having relatives living nearby. Three women reported having an ID Poor card (a designation indicating free or subsidized access to health services due to socioeconomic status), while the majority (n = 12) did not. Although eleven women reported not using a form of contraceptives, two were currently using contraceptives, while two additional women reported planning to use contraceptives in the future.

**Table 1:** Characteristics of participants–Mothers (n = 15).

		n
**Age, in years**		
	< 25	5
	25–30	8
	> 31	2
**Education, in years**		
	<5	3
	5–10	10
	>10	2
**Occupation**		
	None	3
	Garment Worker	4
	Multiple Occupations	5
	Other	2
	N/A	1
**Health Center**		
	Leay Boh	3
	Roveang	3
	Prambay Mum	2
	Roka Krau	1
	Tonle Bati	1
	Saman	1
	Angkor Borey	1
	Prambay Mum	1
	Phung Tram	1
	N/A	1
**Religion**		
	Buddhist	15
**ID Poor Card**		
	Yes	3
	No	12
**Relatives/family living nearby**		
	Yes	15
	No	0
**Contraceptive Use**		
	Yes	2
	No	11
	Plans to use	2

Analysis resulted in exploration of the following themes: valuing of care through different lenses, transitioning forms of postnatal care, and diverse sources of advice on postnatal care.

Participants described the value of postpartum care through the lenses of traditional knowledge of the pregnancy and postpartum period, and as also a mechanism to prevent future ill-health. Sub-themes included post-partum vulnerability, common post-natal illnesses or health conditions, and preventive practices.

### Valuing of postnatal care through different lenses

#### Post-partum vulnerability

Postpartum vulnerability was a key sub theme of interviews. The period of pregnancy and post-partum are times when a woman’s body is considered susceptible and at high risk of injury or illness. Women reported that traditional practices were used to both strengthen the mother and to prevent negative health outcomes that could result. *Ang pleung* or “roasting” was a commonly used practice historically to accomplish these two tasks, however, many women described it as outdated. Women’s perceptions towards postpartum vulnerability are illustrated in the below quotes:

*I am vulnerable after delivery*, *like the baby during the first month after delivery*. *This is true especially if the weather is too hot or too cold*. *(Mother*, *23 years old*)

*Chha-an sor sai [heating the body] will make health strong*. *This is why you must do injections or ang pleung*. *(Mother*, *29 years old*)

Due to the vulnerability inherent in the postpartum state, participants reported that they needed some form of care to make sure they were protecting themselves and healing.

*Because I am not allowed to ang pleung*, *I know I would not be healthy again without injection*. *We used to think we need to ang pleung and drink traditional medicines*, *but we can’t do that*. *So*, *we must do injections*. *(Mother*, *35 years old*)

*[My] mom told me I had to do ang pleung or injections*, *and this was told by older generations that you must do this after delivery*. *I don’t know what would happen if someone didn’t do ang pleung or injections after delivery*, *it is a requirement that you must do*. *(Mother*, *23 years old*)

#### Local descriptions of common postnatal health conditions

Participants universally described common ailments that occur after a woman delivers her baby, and the symptoms and conditions associated with these. These included *sor sai kchey*, describing that the whole body is weak, breast milk production issues, and various bodily aches or pains.

*Right after the delivery I felt weak*, *and after the injections I think that I’m strong like before the delivery*, *like normal before delivery*. *(Mother*, *23 years old*)

One illness condition that was commonly described across participants was *toa*, which postpartum women sought to avoid through the use of preventive practices.

*The symptoms of [toa] is getting stomach aches*, *and feeling “wind” in the stomach*. *The skin would not be bright*, *and they would become skinny*. *This type of illness can’t be cured*. *Our skin will become like skin of the serpent or scale of the fish*. *Darker skin and skinnier*. *(Mother*, *26 years old*)

#### Preventive practices

*Nutrition and food*. Diet was noted as an essential component of recovery from giving birth. A common theme from several of the interviews was that what one ate was a determinant of if, and how well, they recovered from childbirth. Appropriate foods for recovery were seen to convey a general feeling of improved health, increased breast milk production, better bodily functions (sweating, urination, and defecation), and a healthy appetite.

*Sweet*, *salty*, *and spicy with black pepper*, *pork and beef*. *I feel the food improved the breast milk production*. *I ate some green leafy vegetables as well*. *This diet 2 and a half months*. *Also hot water with herbs*, *and I liked the taste*. *It also made me want to eat more*. *(Mother*, *30 years old*)

*I boiled the green leafy vegetable with pork ribs*. *I was eating all different types of meat*, *but all boiled and not fried*. *Eating these meats helped me urinate and defecate well*. *At first*, *it was tough to urinate*, *but after I ate the meat with the clear soup*, *it became better*. *(Mother*, *23 years old*)

This time she ate very carefully, while last time she didn’t. (Mother, 31 years old)

*No one from the health center asked us to eat [heat-producing foods] and they told us to eat everything*, *but we wanted to be healthy*. *(Mother*, *26 years old*)

An issue with obtaining special foods thought to aid in recovery from childbirth was that these were usually expensive, and costly for women to buy. In these situations, they would continue to consume their typical or locally grown food. Although women mentioned desiring special foods, the interviewees mentioned that they also felt their normal food did help them to heal.

*I can’t eat the special food*, *because I can’t afford it*, *so I tried to eat a little bit of the regular food*. *If it didn’t upset my stomach*, *I would continue eating it*.

*(Mother*, *23 years old*)

*The coordinator came to tell her to eat foods that grew locally*, *and not worry about eating the expensive foods*.*(Mother*, *24 years old*)

*Herbal and alcohol tonics*. Herbs were often consumed through a boiling process or in tonic form with wine. Beeswax was also noted as an ingredients in tonics. Participants reported believing that consumption of tonic or rice wine was effective in improving health, healing, and increasing breast milk. This particular behavior was noted as belonging to an older generation, and was thought of as a traditional approach to postnatal care. Herbs were stated to cost approximately 30,000 riel ($7–8) and address various postnatal health issues. Some illustrative descriptions are below.

*I boiled herbs with water*, *which helps with the chha-an sor sai*, *and with production of breast milk*. *(Mother*, *27 years old*)

*I felt that toa has affected my voice till today*. *There weren’t any formal medical personnel to go to*, *so I went to a traditional healer*. *The healer gave water and herbs and helped*, *people thought I was going to die*. *I’m thankful to the healer to this day*. *(Mother*, *30 years old*)

*I also get more breast milk after boiling the root of a tree*. *We drink it hot*. *I drink this from the first day after delivery until today*. *The bag of tree root cost 30*,*000 riel [approximately $7*.*50]*.

*(Mother*, *31 years old*)

*I drink these because I am following the older generation*. *I feel like I want to eat more after drinking these*, *even today*. *It also helped my body feel “cooked*,*” chha-an sor sai*. *Drinking these also prevents toa*, *no matter what you eat*. *It also prevents back and leg pain*, *along with general body ache*. *Even without modern medicine*, *this can greatly improve our health*. *(Mother*, *36 years old*)

Tonics, including those with alcohol, were consumed by some mothers, but others avoided them. One woman stated:

*I didn’t drink the special wine*. *I never drank wine or alcohol before*, *so I think that I can’t support it*. *I don’t feel well after drinking alcohol*. *(Mother*, *25 years old*)

*Application of heat or cold to the abdomen*. Ice blocks or hot stones (used singly, not in combination) applied to the abdomen were described by some participants as helpful to postpartum health. The interviewees who spoke of this practice noted it could slim one’s stomach and ease stomach pains.

*My mother would put a hot stone on my stomach in the morning*. *This makes me slim again*, *as my belly expanded a lot during pregnancy*. *(Mother*, *23 years old*)

*I didn’t do the stone but did put one block of ice on the stomach*. *I felt better after she did this*, *and it wasn’t uncomfortable*. *This took place for 4–5 days*, *and they put the ice in a plastic bag*, *and then on the stomach*. *They would leave it on until it melted*. *This cost 2*,*000 riel a day*. *(Mother*, *26 years old*)

*The hot stone helps with the pain in the stomach because right after delivery there is so much swelling*, *so it helps to restrict the stomach*. *(Mother*, *27 years old*)

*Steaming*. Steaming is a traditional technique described by some participants utilized specifically for releasing impurities, “bad blood”, or other issues. This postpartum practice was identified as a practice of the older generation, and one suggested by older people in the village. Participants described the following:

*I steam whole body with steam from a clay pot*, *which has citrus*, *salt and alum [aluminum sulfate]*. *The steam brings out the bad blood from the uterus*, *and the bad smell*. *(Mother*, *24 years old*)

*Inside the steaming water*, *I took bamboo and orange leaves*, *and covered my head with a pillow case while I steamed my face*. *I think the steam will help cure the dark blotches on my face*, *which had appeared since my first baby*. *The older people in the village told me about this*. *(Mother*, *27 years old*)

*Application of herbal substances to the skin*. Topical treatments were often used by new mothers to aid in their healing process. Women interviewed described the process of making the mixture, how to utilize it, and what costs were associated. “Yellow powder” or ground turmeric, and rice wine mixture was utilized as a topical salve. Yellow powder was utilized to make the woman feel “warmer” and to improve the skin of the mother after delivery. Costs ranged from 15,000 to 100,000 riel procure these substances.

*I put yellow powder all over my body for 15 days*. *I got it from the market and ground it by myself*. *It cost 15*,*000 riel for 1 kg*. *I mix it with homemade rice wine*, *and put it on in the morning after bath*, *and wash it off before I sleep*. *The yellow powder keeps skin brighter later in life and keep me warm as well*.

*(Mother*, *30 years old*)

*Importance of traditional practices following delivery*. Traditional medicine was highly regarded by those interviewed. These practices were thought of as ones passed down through generations. Although utilization of these techniques was common, they were not necessarily reported as being done in place of modern medicine. Women reported that they would do these practices (steaming, tonic, skin application) as a compliment to their postnatal care, as finances allowed. Women reported receiving injections and modern medicine while using these traditional techniques. These practices were thought to help with postpartum vulnerability. When speaking of these traditional practices, women stated:

*We follow these practices because our ancestors did*. *My great grandmother did the same thing with my grandmother*. *(Mother*, *26 years old*)

*I drink these because I am following the older generation*.*(Mother*, *27 years old*)

### Transitioning forms of care

The theme of transitioning forms of postnatal care was related to moving away from traditional forms of care, or alternatively, mixing traditional and modern health care practices. Participants described forms of care that were more commonly practiced due to modern availability and perception of effectiveness, including injections.

#### Heat injections

The traditional practice of *ang pleung* has been discouraged due to potential danger for women and newborns. Participants reported that health center staff and midwives advise against *ang pleung*. Medical injections were suggested as an alternative and appeared to be very common in the study area.

*The people at the HC told her she can’t ang pleung*, *so they recommended injections*. *I feel that I will be healthy later in life if I get injections*, *but on this I am not sure*. *(Mother*, *29 years old*)

*I used to hear about roasting done a lot*, *but not so much anymore*. *The midwives are strongly against it*, *and so is everyone else*. *(Mother*, *37 years old*)

*The injections made me feel… normal*, *just like us*. *The body became weak after pregnancy*. *(Mother*, *29 years old*)

*I plan to have one more child*, *maybe in three years*. *I will do the same injections next time*. *I was thinking about ang pleung but everyone says it is not allowed*, *so the only thing left is injections*. *(Mother*, *24 years old*)

Several women stated that they were relieved not to practice *ang pleung*, and spoke of how uncomfortable the experience was. Several other women acknowledged that there was a movement away from *ang pleung*. Utilization of injections in their place had come to be considered normal.

*I couldn’t do roasting*, *so did injections*. *When I delivered my first baby*, *I did ang pleung*, *but couldn’t bear to be in a room that was so hot and had so much smoke*, *so [this time] I did injections*. *(Mother*, *23 years old*)

*There is no roasting practiced in this community anymore*. *(Mother*, *27 years old*)

*The midwife told me that because I had an episiotomy I can’t do ang pleung because the stitches shouldn’t be heated as they will dissolve by themselves*. *I didn’t want to do ang pleung*. *Nowadays*, *most people don’t do ang pleung*. *The injections work just as well for chha-an sor sai*. *(Mother*, *24 years old*)

*I did roasting*, *but now the injections are the things that people do*. *(Mother of an interviewee*, *Grandmother*)

*In the older generation*, *we used roasting to get warm*, *but since roasting isn’t allowed*, *we use injections now…I feel warm from the end of my fingertips to my whole body*. *I think being hot protects me against disease*. *(Mother*, *31 years old*)

The regimen of injections varied across interviews. Some women had injections for several days, and some women had injections for two weeks. Women were given injections as often as two times a day to four times a day. Women reported that the injections were given by the midwives from the community. Injections reportedly took place mainly in the woman’s home. Some women stated that their midwife suggested, or offered, the injections. Other women stated that they had to request the service from the midwives.

*I asked the midwife to come and visit me at home privately and give injections*, *and then I gave her money*. *I had injections for seven days*, *two times a day*, *with three injections each time in the hips*. *It cost 230*,*000 riel total*. *(Mother*, *29 years old*)

*The midwife from the health center gave me injections (chak thnam) because I asked her*. *It is a must after delivery*. *We couldn’t do ang pleung*. *(Mother*, *23 years old*)

*The injections made me feel warm*. *I don’t exactly know what was in them*, *but I took them once a day for two weeks…the total cost was over 200*,*000 riel*, *and I paid the money at the end*. *(Mother*, *30 years old*)

*We paid 430*,*000 riels for four injections for 12 days*. *They used four syringes with two needles every day for 12 days (48 total injections)*. *There were two injections in the arm and two in the thigh*. *(Mother*, *25 years old*)

#### Physical reactions to injections

Overall women reported that the injections helped them to feel better. A desired component of was the feeling or sensation of warming of the body. This warming sensation was a key component of the injections sought by participants. In addition to warmth, women experienced increased appetite, more breast milk, better sleep, less pain, and more energy. The interviewees also described that the injections helped with getting rid of “bad blood” and uterine contractions. Women stated:

*I felt I had more breast milk and could eat more food after injections*. *(Mother*, *30 years old*)

*I didn’t know what was inside the injections just that they made me warm*. *I feel better and better and better [after the injections]*. *I think that the injections may help me be stronger and I won’t get any diseases…When I got injections*, *I felt warm*, *so I think they [the injections] helped get rid of bad blood*. *(Mother*, *25 years old*)

*I decided to get injections again because it would reduce the pain*. *I feel like when I get injections*, *my body feels normal*. *(Mother*, *29 years old*)

*After the injection*, *I had more of an appetite and I slept better*. *(Mother*, *30 years old*)

*I felt pain in my back before the injections*, *and felt better after the injections* …*I believe that after delivered my first boy and got injections I felt I became as I was before the first pregnancy—able to work hard*. *I felt this way*, *and did it this time*. *(Mother*, *29 years old*)

Attitudes towards injections were overall very positive. Not doing injections or heating were associated with not feeling well, or not healing well. Women reported not wanting to do *ang pleung* and wanting to do the injections instead because heating was necessary. Several interviewees stated that injections allowed them to recover quickly and to get back to feeling normal. The injections were also viewed as easier than other traditional practices.

*The injections are modern medicine and it’s easier to do*. *With the powder and things*, *that’s a lot of things to do*. *(Mother*, *35 years old*)

*I feel the injection would get rid of the bad blood in [my] uterus*, *and also because the midwife would come*, *she could check on the vaginal tear every day*. *The injections make the bad blood come out faster*. *(Mother*, *23 years old*)

*So*, *when we have a baby*, *we feel numb in our hands and our legs*, *and after receiving the injection we just feel normal*, *as if we didn’t have the baby*, *like a normal healthy person*. *(Mother*, *25 years old*)

Although the injections were mostly perceived as positive within the community, there were some issues such as cost and perceived efficacy. Reported cost of injections varied from participant to participant, ranging from 120,000 riel to 430,000 riel [$29.58 to $106]. This cost appeared to be be based on quantity of injections and specific types. Difficulty finding the funds to cover injections, which are all out of pocket and privately arranged, was mentioned by participants. Interviewees stated:

*I had injections*, *the first time for five days*, *but this time for three days*, *as I didn’t have enough money to pay*. *(Mother*, *23 years old*)

*The midwife told me to get injections*, *but I couldn’t afford them*, *so I just used traditional medicines*. *(Mother*, *23 years old*)

There was a component of uncertainty around the effectiveness of injections or whether there were side effects. Some interviewees stated that they were unsure of whether a positive heath outcome could be associated with the injections, or with other factors. There were also some health issues that could not be changed by injections. Quotes illustrating these concepts are presented below.

*After [the injections] the breast milk came out more*, *but I’m not sure if this is from diet or the injections*. *(Mother*, *30 years old*)

*The tonic drinks are the major thing that prevents*, *sboan loan [the sound of the uterus/ prolapse]*. *The injections don’t do much for this*. *(Mother*, *27 years old*)

*A small pustule appeared on the baby’s head*, *and I asked the midwife to look at it*. *The midwife said it was because she gave injections which caused the baby to have the pustule as the injection could have transmitted through the breastmilk*, *and that it would go away in two or three days*. *(Mother*, *29 years old*)

*There is no problem for the health if injections are not done*. *Injections don’t help the baby*. *The injections help the mother’s vaginal recovery*. *(Mother*, *30 years old*)

### Sources of advice

A major theme throughout the interviews was sources of advice and associated communication on postnatal care. Participants reported that their postnatal care practices were influenced by advice given to them by trusted individuals. Women reported family members, elders in the community, and healthcare providers as being influential individuals. Interviewee’s mothers and midwives were the most frequently reported to offer advice. These individuals were also deemed knowledgeable on the topic of maternal and child health. Interviewees stated:

*I did injections for my previous births as well*. *The midwives advised me not to do ang pleung*. *(Mother*, *27 years old*)

*The midwife mentioned that this generation should get injections*. *Usually everyone gets injections*. *It is common knowledge*! *(Mother*, *31 years old*)

*Women in this village do not only the injections but every woman in the village does whatever is told by the Health Center staff*, *not what the family says*. *Like the lotion to apply on the umbilical cord*, *before they didn’t use to use that*, *but now they do*. *(Mother*, *31 years old*)

*I asked the midwife to come to give injections because I didn’t want to do ang pleung*. *My older sister didn’t do ang pleung*, *so because my older sister didn’t do that I followed her example*. *(Mother*, *29 years old*)

## Discussion

This ethnographic research sought to query and describe traditional and modern postnatal care practices as understood and practiced by women who have recently given birth, since these have continued to evolve over time. This was accomplished through in-depth interviews and unstructured observation with mothers who had given birth in the last 6 months. Themes from the data analysis related to transitioning forms of postnatal care, valuing of care through different lenses, and diverse sources of advice on postnatal care were identified, along with a focus on medical injections.

The findings of this research confirmed those from previous studies regarding the use of traditional methods of care for Cambodian women in the perinatal period [9, 11, 13–14]. Uniquely, our study identified injections as an important, and indeed customary, component of the postnatal care regimen described by participants, which had previously only been mentioned in passing by one peer-reviewed study citing data collected in 1995 without reference to injections for heat [33], and one student report of an interview study from 2008 [13]. The study by White mentioned injections without any specific detail, as one among many practices that women in Cambodia used primarily to ensure that “bad blood” from pregnancy was expelled in the post-partum period [33], while the work by Montesanti described “heat injections” [13].

A broader, recent review of traditional beliefs and practices during the perinatal period in Asian countries identified many similar themes to those noted here, including hot/cold balance, dietary modifications, and the importance of protecting future health by enacting preventive practices during pregnancy and the postpartum period, however, other than for a brief mention of the study noted above, which does not provide detail on the topic, the recent comprehensive review notably did not identify injections to promote heat in the body as a reported in the literature from any other source [21]. Generally among studies from the region, preventive care during the post-partum period is expected to protect later health, and practices for keeping the body warm feature prominently, along with dietary modifications, and rest [34, 35]. In a study from neighboring country Myanmar, the authors report many post-partum practices similar to those identified in this study, including a focus on warming the body through internal or external means such as diet and application of “warming” substances such as turmeric, respectively [36]. Another study from Myanmar identified inducing perspiration through steam or other means among postpartum practices performed by women, similar to the practice identified here [37]. Sharma described traditional perinatal care practices in Nepal, which differed somewhat from those reported in this study barring rest and seclusion, and noted that “local people’s beliefs and practices offer both opportunities and barriers to health service providers…” concluding that “maternity care providers need to be aware of local values, beliefs and traditions to anticipate and meet the needs of women, gain their trust and work with them” [38].

Similarly, this study also provides insight into what kind of post-partum health care community members want to receive, often described as the demand-side of the health services. A study by Dingle et al. noted that although equity gaps were improving in use of reproductive and maternal health services in Cambodia, postnatal care was one of the least utilized health services, and additionally noted a slight increase in inequity from 2000 to 2010 [7]. Previous qualitative work has described barriers to postpartum care seeking care including: a need to stay in the house for healing, a need to tend to household duties, or not having the autonomy to make decisions about care seeking [13], but it is unknown whether or how injections might hinder care. White noted that “midwives often serve a cultural brokers interpreting the culture of professional midwifery with its biomedical beliefs to women whose cultural beliefs about pregnancy and birth may be significantly different” [39]. This role may be critical in ensuring appropriate and high-quality care, and future research in Cambodia should address the extent of the practice of injections for post-partum care, the specific manner in which these are provided, the medications used, and how these may impact individual women’s health.

Based on review of the literature, this is the first study providing detailed description of the use of injections for postnatal health care in Cambodia and the cultural etiologies underlying the practice, which appears to be very common based on media reports and available information [20, 21]. In light of a 2015 HIV outbreak that occurred in rural Cambodia due to unsafe injection practices by a private health provider, affecting 242 individuals including mothers and children, this issue is particularly relevant [40]. While it is not reported whether post-partum injections for heat were part of the private provider’s practice in question, the case demonstrates the level of risk and potential for harm reduction by investigating this topic. Given the possible health dangers associated with unnecessary medical injections in Cambodia, a recent intervention sought to develop an educational tool to improve population level understanding of injections as a medical treatment [15]. The authors noted that knowledge about injection safety was increased following intervention, and that such improvement in knowledge would be a helpful strategy to scale up. The data presented here may be used to implement a similar strategy through understanding the importance of traditional and cultural explanations around postnatal injections.

What has been learned from the study may be used by others to improve perinatal care at both individual and population level. The key results of this research, namely that women in this setting and similar Southeast Asian settings prefer to adhere to traditional customs regarding maintaining warmth in the body during the post-partum period and rely on traditional postnatal practices for ensuring future health, can be used to inform recommendations.

A key recommendation is that health care providers and population health programs align postnatal care recommendations with culturally important beliefs. For example, the postnatal care recommendation that iron and folic acid supplementation should be provided for at least 3 months after delivery could be aligned with the message that care in the postpartum period impacts future health [41]. Another recommendation is that the guideline for home visits to assess postpartum health [42] be aligned with cultural preferences for staying warm and remaining in the home postpartum.

An additional recommendation is that where post-partum injections for heat are desired, other less potentially harmful practices are suggested through social behavior change messaging and health care workers. For example, suggesting intake of warm water in conjunction with recommended iron and folic acid supplements may address the desire to keep the body warm and still follow essential care practices. In this way, it may be possible to adhere to traditionally important practices while maintaining recommended postnatal care guidance.

Finally, given that traditional postpartum practices are passed on through older family members, it is recommended that maternal health counselling throughout the continuum of care include not only the mother, but also close family relatives involved in supporting mother and newborn, such as grandmothers and aunts. [21] Counseling and education on maternal care practices and educational programs should be inclusive of not only women but also husbands, parents, and in-laws. This may include discussion of traditional practices, such as warming the body, and how these could impact health.

Strengths of this study include the questions answered regarding what postpartum practices are used and why, the specificity of focus provided from in-depth interviews and ethnographic context, and insights gained. Our data add new information to the peer-reviewed literature on postnatal care in Cambodia concerning use of medical injections, which come with attendant potential health consequences. As a widespread practice, this would be important to the overall landscape of maternal and neonatal health in the country. However, the study has limitations given the ethnographic nature, thus this information is not intended to be widely generalizable. The results of the study may be utilized as a background for future quantitative studies and interventions to improve maternal and newborn health.

In order to continue the trend toward improving accessibility and quality of postnatal care in the country for sustained improvement in maternal and newborn health outcomes [43], health care workers and policy makers must take into account how traditional understandings of health inform current care practices. In future, it will be important to utilize the understanding provided by this research on sociocultural dimensions of postpartum health and illness to address both demand and supply side issues in the provision of postnatal health services.

## References

[1] Bhutta Z, JK D, Bahl R, Lawn J, Salam R, Paul V, et al Can available interventions end preventable deaths in mothers, newborn babies, and stillbirths, and at what cost? Lancet. 2014;384(9940):347–70. 10.1016/S0140-6736(14)60792-3 24853604

[2] Salam RA, Mansoor T, Mallick D, Lassi ZS, Das JK, Bhutta ZA. Essential childbirth and postnatal interventions for improved maternal and neonatal health. Reprod Health. 2014;11 Suppl 1:S3. 10.1186/1742-4755-11-S1-S3 PMC4145857 25177795

[3] World Health Organization. Success Factors for Women’s and Children’s Health:Cambodia. Geneva, Switzerland2015.

[4] Kikuchi K, Yasuoka J, Nanishi K, Ahmed A, Nohara Y, Nishikitani M, et al Postnatal care could be the key to improving the continuum of care in maternal and child health in Ratanakiri, Cambodia. 2018;13(6)::e0198829 10.1371/journal.pone.0198829 29889894 PMC5995361

[5] National Institute of SC, Directorate General for HC, International ICF. Cambodia Demographic and Health Survey 2014. Phnom Penh, Cambodia: National Institute of Statistics/Cambodia, Directorate General for Health/Cambodia, and ICF International; 2015.

[6] Woods J, Gagliardi L, Nara S, Phally S, Varang O, Viphou N, et al An innovative approach to in-service training of maternal health staff in Cambodian hospitals. Int J Gynaecol Obstet. 2015;129(2):178–83. 10.1016/j.ijgo.2014.10.034 25593108

[7] Dingle A, Powell-Jackson T, Goodman C. A decade of improvements in equity of access to reproductive and maternal health services in Cambodia, 2000–2010. Int J Equity Health. 2013;12:51 10.1186/1475-9276-12-51 23837577 PMC3723953

[8] Wang W, Hong R. Levels and determinants of continuum of care for maternal and newborn health in Cambodia-evidence from a population-based survey. BMC Pregnancy and Childbirth. 2015;15(1). 10.1186/s12884-015-0497-0 PMC4371879 25885596

[9] Liljestrand J, Sambath MR. Socio-economic improvements and health system strengthening of maternity care are contributing to maternal mortality reduction in Cambodia. Reprod Health Matters. 2012;20(39):62–72. 10.1016/S0968-8080(12)39620-1 22789083

[10] Bigdeli M, Annear PL. Barriers to access and the purchasing function of health equity funds: lessons from Cambodia. Bull World Health Organ. 2009;87(7):560–4. 10.2471/BLT.08.053058 19649372 PMC2704035

[11] Ministry of Health, Kingdom of Cambodia. Fast Track Initiative Road Map for Reducing Maternal & Newborn Mortality. 2010.

[12] White PM. Crossing the river: Khmer women's perceptions of pregnancy and postpartum. J Midwifery Womens Health. 2002;47(4):239–46. 10.1016/s1526-9523(02)00266-0 12138931

[13] Montesanti S. Cultural Perceptions of Maternal Illness among Khmer Women in Krong Kep, Cambodia (PDF Download Available). Explorations in Anthropology. 2011;11(1):90–106.

[14] Bazzano AN, Taub L., Oberhelman R.A., Var C. Newborn Care in the Home and Health Facility: Formative Findings for Intervention Research in Cambodia. Healthcare (Basel). 2016;4(4). 10.3390/healthcare4040094 28009812 PMC5198136

[15] Ith P, Dawson A, Homer CS, Klinken Whelan A. Practices of skilled birth attendants during labour, birth and the immediate postpartum period in Cambodia. Midwifery. 2013;29(4):300–7. 10.1016/j.midw.2012.01.010 22342172

[16] Ith P, Dawson A, Homer CS. Women's perspective of maternity care in Cambodia. Women Birth. 2013;26(1):71–5. 10.1016/j.wombi.2012.05.002 22722014

[17] Health Mo. Health Service Delivery Profile Cambodia. Phnom Penh, Cambodia2012.

[18] Ros B, Le G, McPake B, Fustukian S. The commercialization of traditional medicine in modern Cambodia. Health Policy Plan. 2018;33(1):9–16. 10.1093/heapol/czx144 29040469 PMC5886242

[19] White P. Heat, Balance, Humors, and Ghosts: Postpartum in Cambodia. Health Care for Women International. 2010;25(2):179–94. 10.1080/07399330490267477 14766432

[20] Turner C, Pol S, Suon K, Neou L, Day NPJ, Parker M, et al Beliefs and practices during pregnancy, post-partum and in the first days of an infant’s life in rural Cambodia. BMC Pregnancy Childbirth. 2017;17 10.1186/s12884-016-1217-0 28403813 PMC5389162

[21] Withers M, Kharazmi N, Lim E. Traditional beliefs and practices in pregnancy, childbirth and postpartum: A review of the evidence from Asian countries. Midwifery. 2018;56:158–70. 10.1016/j.midw.2017.10.019 29132060

[22] Ozawa S, Villar-Uribe M, Evans DR, Kulkarni V, Ir P. Building informed trust: developing an educational tool for injection practices and health insurance in Cambodia. Health Policy Plan. 2018;33(9):1009–17. 10.1093/heapol/czy080 30312416

[23] Janssens F. Birth, wind and fire. South East Asia Globle. 2012.

[24] Vachon M. Traditional Post-Birthing Practice May Endanger New Mothers and Children. The Cambodia Daily. 2004.

[25] Ozawa S, Yemeke TT, Tawah AF, Kulkarni V, Villar Uribe M. Out-of-Pocket Household Expenditures on Medical Injections in Cambodia. Pharmacoecon Open. 2018. 10.1007/s41669-018-0067-2 PMC6249188 29427148

[26] Hoban E, Liamputtong P. Cambodian migrant women's postpartum experiences in Victoria, Australia. Midwifery. 2013;29(7):772–8. 10.1016/j.midw.2012.06.021 22882970

[27] Kermode M. Unsafe injections in low-income country health settings: need for injection safety promotion to prevent the spread of blood-borne viruses. Health Promot Int. 2004;19(1):95–103. 10.1093/heapro/dah110 14976177

[28] Palinkas LA, Horwitz SM, Green CA, Wisdom JP, Duan N, Hoagwood K. Purposeful sampling for qualitative data collection and analysis in mixed method implementation research. Adm Policy Ment Health. 2015;42(5):533–44. 10.1007/s10488-013-0528-y 24193818 PMC4012002

[29] Guest G, Bunce A, Johnson L. How Many Interviews Are Enough?: An Experiment with Data Saturation and Variability. Field Methods. 2006;18(1):59–82.

[30] Var C, Bazzano AN, Srivastav SK, Welty JC, Ek NI, Oberhelman RA. Newborn Infection Control and Care Initiative for health facilities to accelerate reduction of newborn mortality (NICCI): study protocol for a randomized controlled trial. Trials. 2015;16:257 10.1186/s13063-015-0771-5 26044715 PMC4458036

[31] Tong A, Sainsbury P, Craig J. Consolidated criteria for reporting qualitative research (COREQ): a 32-item checklist for interviews and focus groups. Int J Qual Health Care. 2007;19(6):349–57. 10.1093/intqhc/mzm042 17872937

[32] Downe-Wamboldt B. Content analysis: method, applications, and issues. Health Care Women Int. 1992;13(3):313–21. 10.1080/07399339209516006 1399871

[33] White P. Crossing the river: Khmer women's perceptions of pregnancy and postpartum. J Midwifery Womens Health. 2004;47(4):239–46. 10.1016/s1526-9523(02)00266-0 12138931

[34] J J. Hmong Cultural Practices and Beliefs: The Postpartum Period. Clinical nursing research. 1995;4(3). 10.1177/105477389500400309 7633342

[35] Lundberg P, TN T. Vietnamese women's cultural beliefs and practices related to the postpartum period. Midwifery. 2011;27(5):731–6. 10.1016/j.midw.2010.02.006 20400214

[36] G S, Y A, AM F. "She Learned it from her Mother and Grandmother": Women's Experiences with Delivery and Post-partum Practices in Peri-urban Yangon, Myanmar.—PubMed—NCBI. Maternal Child Health J. 2016;20(4):854–61. 10.1007/s10995-016-1918-z 26880487

[37] KK S. Beliefs and practices surrounding postpartum period among Myanmar women. Midwifery. 2013;29(11):1257–63. 10.1016/j.midw.2012.11.012 23415368

[38] Sharma S, van Teijlingen E, Hundley V, Angell C, Simkhada P. Dirty and 40 days in the wilderness: Eliciting childbirth and postnatal cultural practices and beliefs in Nepal. BMC Pregnancy Childbirth. 2016;16(1):147 10.1186/s12884-016-0938-4 27381177 PMC4933986

[39] White PM. Heat, balance, humors, and ghosts: postpartum in Cambodia. Health Care Women Int. 2004;25(2):179–94. 10.1080/07399330490267477 14766432

[40] Rouet F, al. e,. Massive Iatrogenic Outbreak of Human Immunodeficiency Virus Type 1 in Rural Cambodia, 2014–2015. Clinical Infectious Diseases. 2017;66(11):1733–41. 10.1093/cid/cix1071 PMC5963970 29211835

[41] Organization WH. Pregnancy, Childbirth, Postpartum and Newborn Care: A Guide for Essential Practice (3rd Edition). Geneva: World Health Organization; 2015 2015. 26561684

[42] World Health Organization. Postnatal care of the mother and newborn. Geneva; 2013. 24624481

[43] Mallick L, Allen C, Hong R. Trends in Maternal and Child Health in Cambodia, 2000–2014 Further Analysis of the 2000, 2005, 2010, and 2014 Cambodia Demographic and Health Surveys. Rockville, Maryland, USA: ICF,; 2016.

